# Application of corneal injury models in dual fluorescent reporter transgenic mice to understand the roles of the cornea and limbus in angiogenic and lymphangiogenic privilege

**DOI:** 10.1038/s41598-019-48811-z

**Published:** 2019-08-23

**Authors:** Xinbo Gao, Kai Guo, Samuel M. Santosa, Mario Montana, Michael Yamakawa, Joelle A. Hallak, Kyu-Yeon Han, Susan J. Doh, Mark I. Rosenblatt, Jin-Hong Chang, Dimitri T. Azar

**Affiliations:** Department of Ophthalmology and Visual Sciences, Illinois Eye and Ear Infirmary, College of Medicine, University of Illinois at Chicago, Chicago, Illinois USA

**Keywords:** Cellular imaging, Molecular biology

## Abstract

The role of the corneal epithelium and limbus in corneal avascularity and pathological neovascularization (NV) is not well understood. To investigate the contributions of the corneal and limbal epithelia in angiogenic and lymphangiogenic privilege, we designed five injury models involving debridement of different portions of the cornea and limbus and applied them to the dual-fluorescence reporter Prox1-GFP/Flt1-DsRed mouse, which permits *in vivo* imaging of blood and lymphatic vessels via fluorescence microscopy. Debridement of the whole cornea resulted in significant hemangiogenesis (HA) and lymphangiogenesis (LA), while that of the whole limbus yielded minimal corneal HA or LA. Following hemilimbal plus whole corneal debridement, corneal NV occurred only through the non-injured aspect of the limbus. Overall, these results suggest that the integrity of the corneal epithelium is important for (lymph)angiogenic privilege, whereas the limbus does not act as a physical or physiologic barrier to invading vessels. In CDh5-CreERT2VEGFR2lox/PGFD mice, conditional deletion of vascular endothelial growth factor receptor 2 in vascular endothelial cells abolished injury-induced HA and LA, demonstrating the utility of this transgenic mouse line for identifying important factors in the process of neovascularization.

## Introduction

The cornea is a reliable model for studying the growth of blood and lymphatic vessels, due to its experimental accessibility and avascular nature. The avascularity of the cornea permits direct observation, induction, inhibition, and quantification of vessel formation compared to baseline^1–6^. Thus, the anterior surface of the eye has been a useful tissue model for elucidating the molecular underpinnings of hemangiogenesis (HA) and lymphangiogenesis (LA) as well as the role of vascular growth in oncological, immunological, and ophthalmic pathologies. The active maintenance of corneal avascularity has been termed “corneal angiogenic and lymphangiogenic privilege”^1^, a physiological process that develops during early morphogenesis of the cornea^2^. Importantly, the absence of lymphatics and blood vessels prevents the entry of immunomodulating and inflammatory cells and factors, enabling extraordinary success in histologically incompatible corneal transplantation^4,7,8^. In fact, the 2-year rejection rate for corneal transplant cases is about 10% in low-risk patients who demonstrate little to no vasculature in the cornea prior to surgery^9^. In contrast, drastically higher rejection rates up to 50% have been reported if the corneal bed is already vascularized^4,10^. Interestingly, it has been reported that blockade of pro-lymphangiogenic factors improves the outcomes of corneal grafts in murine models by suppressing up to 75% of lymphangiogenesis in the cornea^1^. Meanwhile, corneal neovascularization (NV), the invasion of growing blood and lymphatic vessels from pre-existing peri-corneal vasculature, represents a major cause of blindness^3,4,11^. Corneal NV is a consequence of several ocular insults, including contact lens-related hypoxia, congenital disorders, chemical burns, limbal stem cell deficiency, allergy, infectious keratitis, autoimmune diseases, and graft rejection, all of which are thought to disrupt the delicate equilibrium between pro- and anti-angiogenic factors^1,3,5,12,13^. The eventual loss of visual acuity can be attributed to edema, persistent inflammation, intrastromal protein and lipid deposition, and scarring made possible by the introduction of blood and lymphatic vessels into the cornea.

The anterior surface of the eye is composed of the avascular corneal epithelium, the conjunctival epithelia containing dense vasculature, and the limbal epithelium between the two^14^. The corneal epithelium is constantly sloughed off and replaced by limbal stem cells that divide and migrate centripetally and anteriorly. Corneal NV is a well-documented consequence of clinical limbal defects or deficiency, a finding that has yet to be explained mechanistically^15^. Currently, it is thought that the limbus may act as a physical and physiological barrier to invading vessels, as well as the migration of conjunctival epithelium into the cornea, during normal corneal homeostasis^16–18^. To investigate the role of the limbus in corneal avascularity and the relative role of corneal factors in suppressing vascular invasion, we generated five distinct injury models that involve debridement of the epithelial layer of various regions, including the limbus, cornea, or both. We then applied these models to our dual fluorescence reporter mice described previously (Prox1-GFP/Flt1-DsRed, or PGFD)^19^. The growth of lymphatic vessels is typically difficult to observe experimentally due to their transparency. However, in PGFD mice, blood endothelial cell expression of DsRed is driven via the Flt1 promoter, and lymphatic endothelial cell expression of green fluorescent protein (GFP) is driven via the Prox1 promoter, allowing for direct, *in vivo* observation of both HA and LA in real-time. Here we report the growth patterns of blood and lymphatic vessels in each of the five corneal and limbal injury models. We also discuss the implications of our results with regards to the role of the limbus and the cornea in maintaining corneal avascularity.

## Materials and Methods

### Prox1-GFP/Flt1-DsRed mice

All animal experiments were done in accordance with guidelines and approved by Institutional Animal Care and Use Committee (IACUC) at the University of Illinois at Chicago. We bred Prox1-GFP mice with Flt1-DsRed mice to generate PGFD mice, in which lymphatic vessels emit green fluorescence and blood vessels emit DsRed fluorescence under Axio Zoom imaging. We used male and female mice (6–8 weeks old).

### Conditional knockout mice

Floxed *VEGFR2* mice (flk1^*fl/fl*^) were obtained from Dr. Thomas N. Sato, Nara Institute of Science and Technology. For conditional deletion of vascular endothelial growth factor receptor 2 (VEGFR2), Flk1^*fl/fl*^ mice were bred with *Tg(Cdh*5-*cre/ERT2)1Rha* and PGFD mice. This resulted in the specific deletion of exon 3 of VEGFR2. For verification, PCR genotyping was used for the VEGFR2 wild-type and mutant alleles, *Cdh5-CreERT2* allele, and PGFD. In these adult mice, VEGFR2 deletion was induced by daily intraperitoneal (IP) injections of 80 mg/kg tamoxifen (Sigma-Aldrich, T5648, St. Louis, MO) in 200 µl corn oil/5% ethanol for 5 days. Mice were kept in individually ventilated cages in pathogen-free conditions. The mice had free access to food and water and were kept in a 12-h light–dark cycle.

### Corneal/limbal injury models

Five experimental debridement models were generated in the eyes of PGFD mice (also summarized in Table 1): (1) debridement of a concentric, circular area of the corneal epithelium (1.5-mm diameter); (2) debridement of the whole corneal epithelium (WC); (3) superficial debridement (no stroma) of the whole limbal plus whole corneal epithelium (superficial L + C); (4) superficial debridement (no stroma) of the whole limbal epithelium (WL); and (5) deep debridement (stroma partially debrided) of half the limbal epithelium and the whole corneal epithelium (HL + C) (Supplemental Fig. 1). Deep debridement of the limbal epithelium in model 5 was confirmed by the lack of limbal blood vessels in the stroma after debridement. These five models were applied to PGFD mice to monitor changes in the vascular profile of the cornea upon injury. Model 2 was also applied to a VEGFR2-knockout mouse (CDh5-CreERT2VEGFR2lox/PGFD) with and without tamoxifen injection to induce conditional VEGFR2 deletion. A corneal rust ring remover (Alger Brush II, Latham & Phillips Ophthalmic, Grove City, OH) was used for debridement^20^.

**Table 1:** Corneal debridement models 1–5 of PGFD mice.

**Prox1-GFP/Flt1-DsRed Corneal Debridement Models (Supplemental Fig.** **1****)**	
Model 1	*Superficial* debridement of a concentric circular area of corneal epithelium (1.5 mm diameter)
Model 2	*Superficial* debridement of the whole corneal epithelium (3.10 ± 0.3 mm diameter)
Model 3	*Superficial* debridement of the whole limbal and corneal epithelium
Model 4	*Superficial* debridement of the whole limbal epithelium
Model 5	*Deep* debridement of the dorsal aspect of limbus plus superficial debridement of the whole corneal epithelium

*Superficial = epithelial debridement, without stromal injury; *Deep = epithelial debridement + stromal injury.

After debridement, the eyes were rinsed with saline, and a topical erythromycin ointment (Bausch and Lomb, Tampa, FL) was applied. Intramuscular injections of 0.1 ml of 0.3 mg/ml buprenorphine (Buprenex; Reckitt Benckiser Healthcare, Hull, UK) were administered post-operation and twice on the following day for pain relief.

The injured eyes were visualized under an Axio Zoom V16 fluorescence microscope (Zeiss Microscopy, Oberkochen, Germany) to assess corneal HA and LA at baseline (before injury) and days 4, 7, 10, and 14 after injury.

### Quantification of HA and LA post-injury

Fluorescence images of mouse eyes from ventral and dorsal views were obtained using a motorized fluorescence stereo zoom microscope (Axio Zoom V16). The microscope was equipped with a 16x zoom with a high numerical aperture of NA 0.25. The images were compiled and analyzed, and the measurements quantified using Adobe Photoshop CS5 image software (Adobe Systems Inc., San Jose, CA). Corneal HA was represented by the percentage of the total corneal area occupied by blood vessels [(area of blood vessels/total cornea area) × 100], and corneal LA was represented by the percentage of the total corneal area occupied by lymphatic vessels [(area of lymphatic vessels/total cornea area) × 100]. Both ventral and dorsal views were imaged pre-injury (baseline) and days 4, 7, 10, and 14 post-injury, and the areas of corneal HA and LA were calculated for mice in each group.

### Statistical analysis

Statistical analysis to compare vessel growth between models was performed using linear mixed models for repeated measures. The trend in vessel growth for the whole corneal epithelial debridement model was used as a control and compared with vessel growth in the other models. The analysis controlled for time and mouse, addressing any effects from correlations between measurements and subject. The plot of residuals was performed for each model to test for normality. All analyses were performed with SAS (SAS Institute Inc., Cary, NC) and R (The R Foundation, Vienna, Austria).

## Results

### Debridement of 1.5-mm-diameter circle in the center of the corneal epithelium

**Figure 1:**
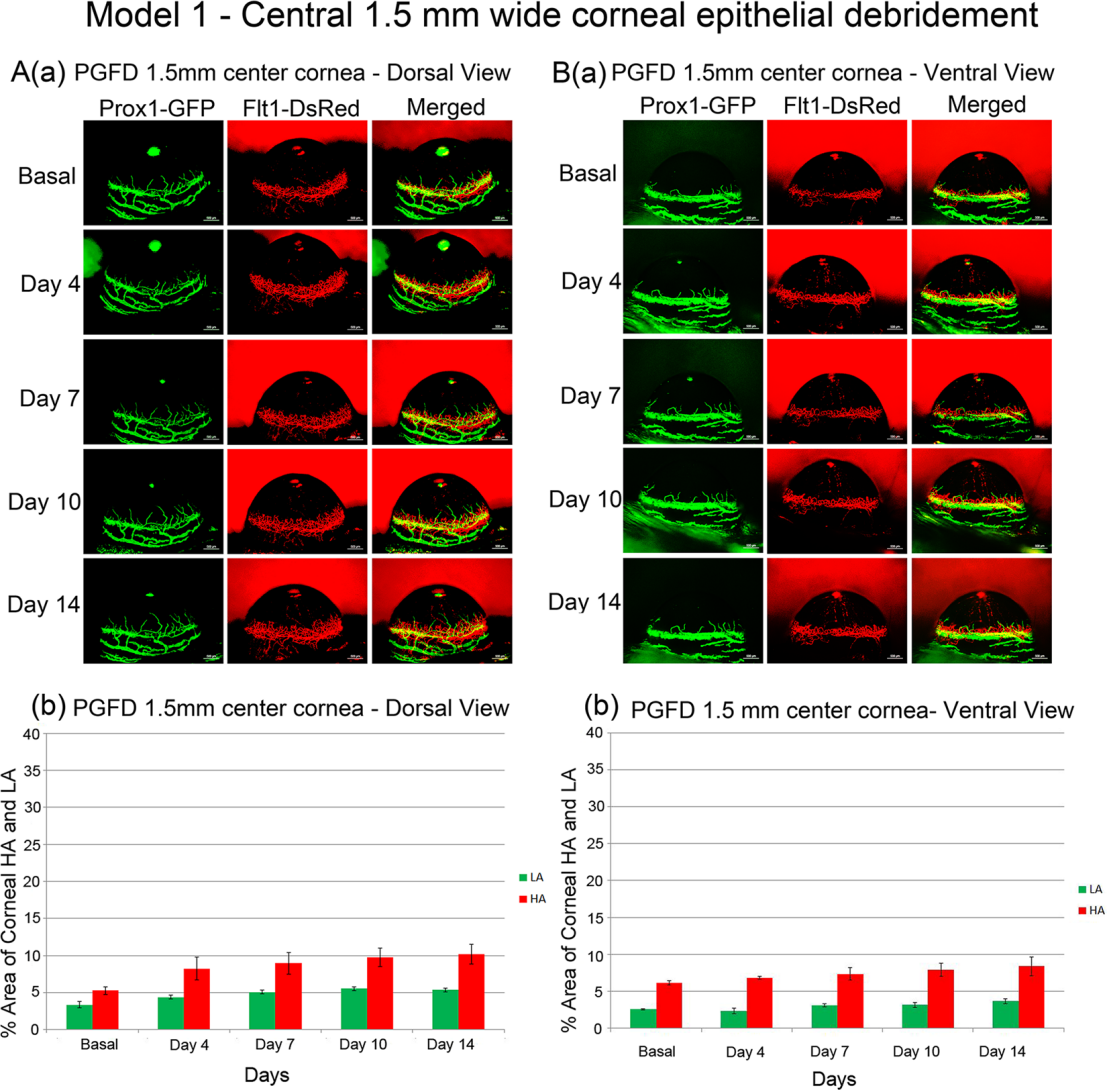
Axio Zoom images of mouse eyes as well as quantitated areas of corneal HA and LA after debridement of a 1.5-mm-diameter circle in the center of the cornea. Visible but minimal neovascularization, particularly HA, was observed. ([**A**] comparison of dorsal HA on day 0 and day 14 (p = 0.0391), comparison of dorsal LA on day 0 and day 14 (p = 0.3831); [**B**] comparison of ventral HA on day 0 and day 14 (p = 0.1887), comparison of ventral LA on day 0 and day 14 (p = 0.4765). *n* = *5*).

In Model 1, a thin layer of a 1.5-mm-diameter area in the central corneal epithelium was debrided (Fig. 1A,B). Dorsally, blood vessel growth was observed by days 10 and 14 and lymphatic vessel growth in the limbus by day 7 and thereafter (Fig. 1A). Ventrally, blood vessels in the limbus showed no change, and lymphatic vessels demonstrated minimal changes by day 14 (Fig. 1B).

### Debridement of the whole corneal epithelium (WC)

**Figure 2:**
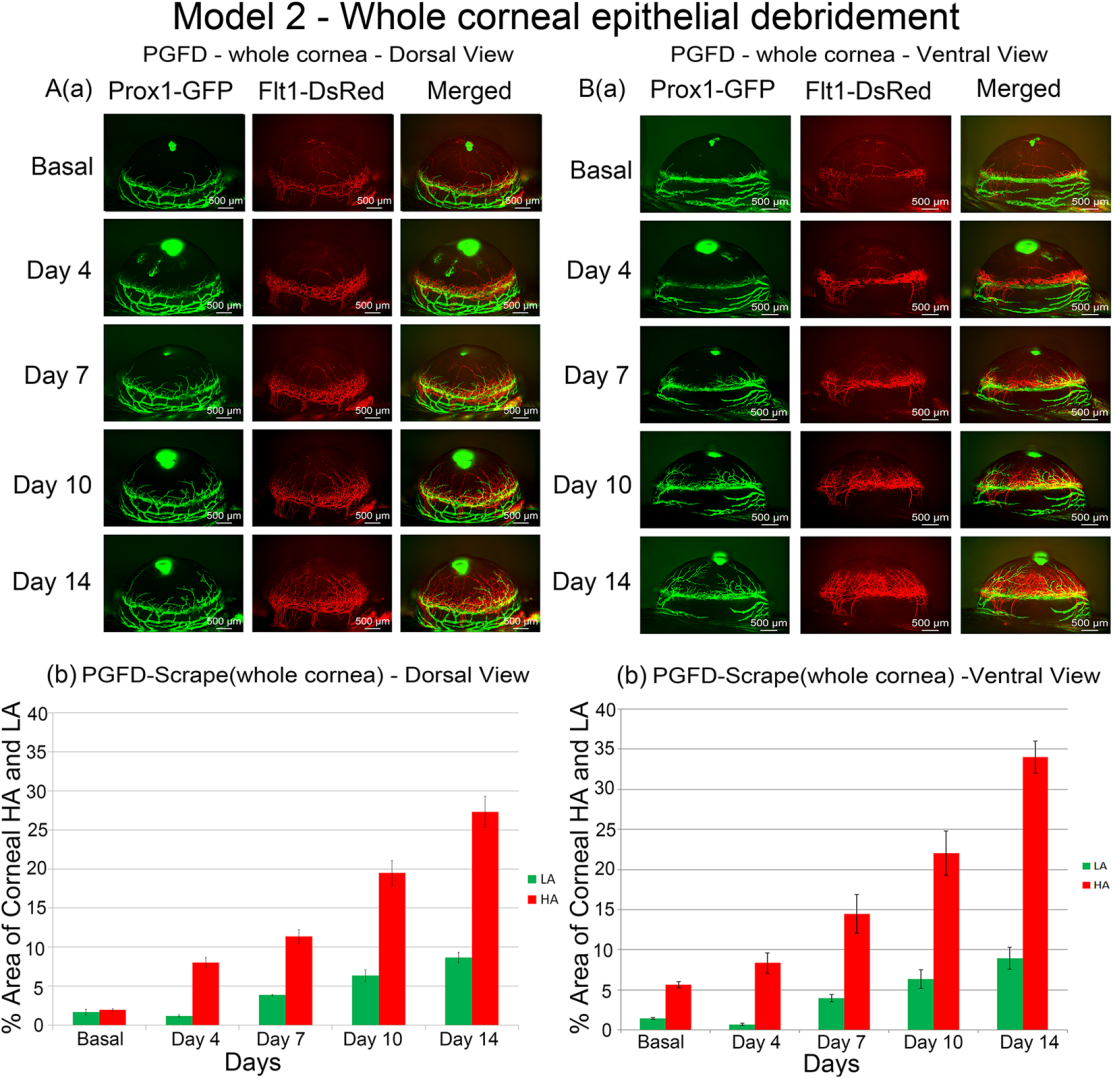
Axio Zoom images of mouse eyes as well as quantitated areas of corneal HA and LA after whole corneal epithelium debridement. Enhanced corneal HA and LA through day 14 were observed from both the dorsal and ventral views. ([**A**] comparison of dorsal HA on day 0 and day 14 (p = 0.0072), comparison of dorsal LA on day 0 and day 14 (p = 0.1698); [**B**] comparison of ventral HA on day 0 and day 14 (p = 0.0198), comparison of ventral LA on day 0 and day 14 (p = 0.2330). *n* = 3).

In Model 2, the whole cornea was debrided and caused significant HA and LA starting from day 4 (Fig. 2). The growth of blood vessels was rapid and surpassed the basal levels by day 7, whereas the growth of lymphatic vessels was significantly more suppressed. However, both blood and lymphatic vessels eventually exceeded their respective baseline levels by day 14. Furthermore, growth rates and vascular densities were comparable between the dorsal and ventral aspects of the cornea.

### Superficial debridement of the whole limbal plus whole corneal epithelium (L + C)

**Figure 3:**
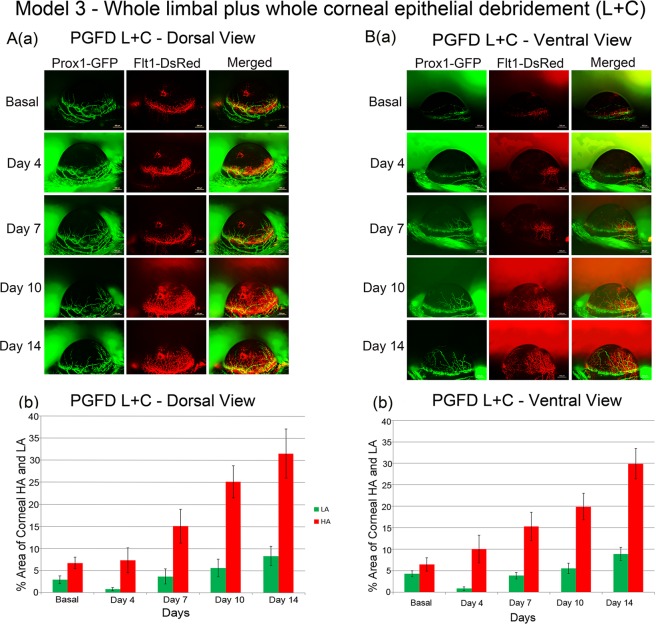
Axio Zoom images of mouse eyes as well as quantitated areas of corneal HA and LA after superficial debridement of the whole limbal plus whole corneal. Enhanced corneal HA and LA through day 14 were observed from both the dorsal and ventral views. ([**A**] comparison of dorsal HA on day 0 and day 14 (p = 0.0014), comparison of dorsal LA on day 0 and day 14 (p = 0.3779); [**B**] comparison of ventral HA on day 0 and day 14 (p = 0.0080), comparison of ventral LA on day 0 and day 14 (p = 0.9251). *n* = 6).

In Model 3, a thin layer of the whole limbal epithelium plus the whole cornea were debrided, sparing the stroma underneath (Fig. 3A,B). From the dorsal view, enhanced HA was seen in the cornea from day 7 and after, while a decrease in lymphatic vessels was seen on day 4 followed by a gradual increase through day 14. Similar growth patterns of blood and lymphatic vessels were observed from the ventral view. Increasing trends in both HA and LA density were seen from the dorsal and ventral views.

### Debridement of the whole limbal epithelium (WL)

**Figure 4:**
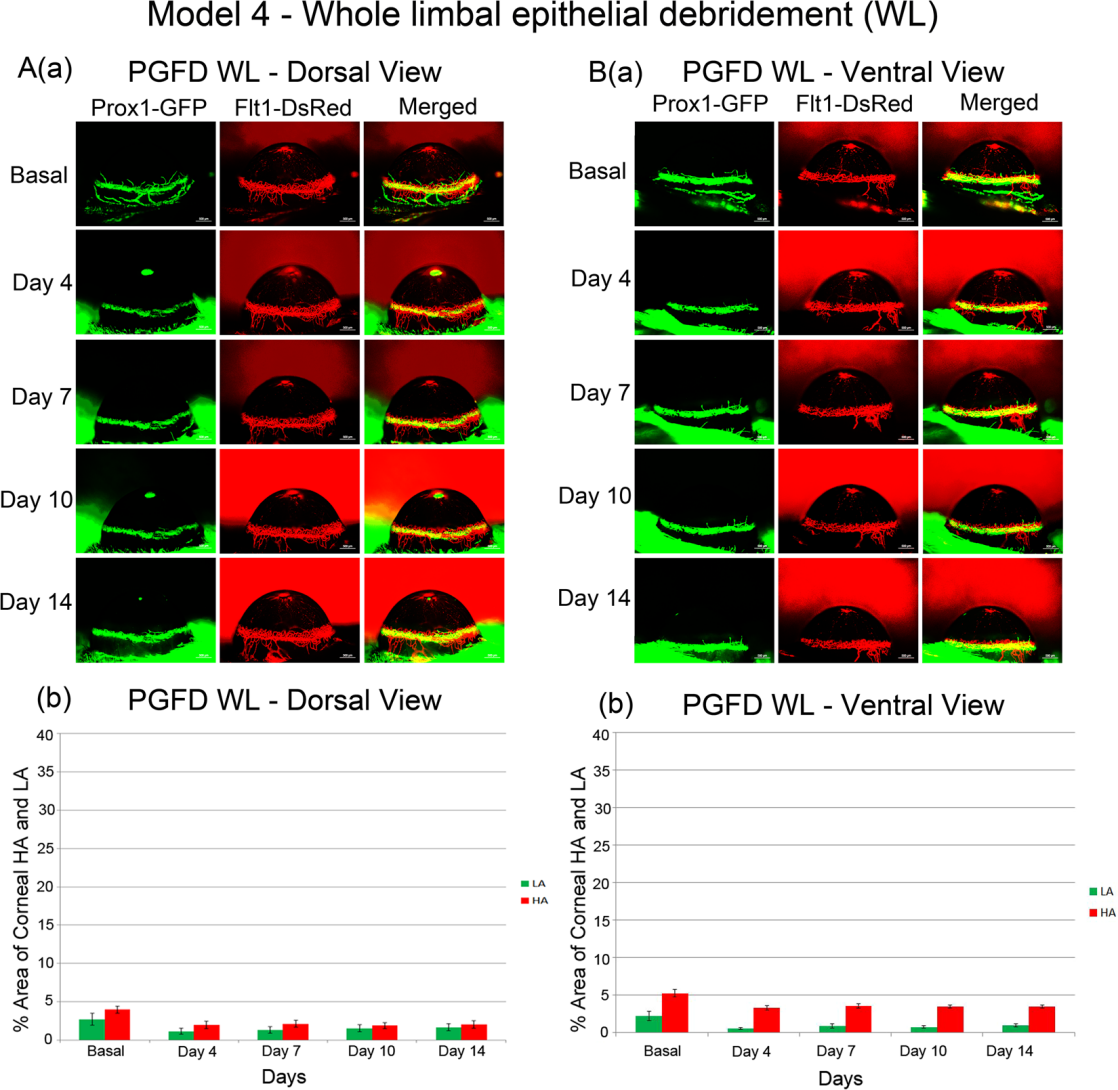
Axio Zoom images of mouse eyes in which the whole limbal epithelium was debrided and quantitated areas of corneal HA and LA. In this model, no discernible corneal HA or LA was observed. ([**A**] comparison of dorsal HA on day 0 and day 14 (p = 0.2412), comparison of dorsal LA on day 0 and day 14 (p = 0.5079); [**B**] comparison of ventral HA on day 0 and day 14 (p = 0.1682), comparison of ventral LA on day 0 and day 14 (p = 0.2740). *n* = 4).

In Model 4, a thin layer of the epithelium of the whole limbus was debrided sparing the stroma underneath (Fig. 4A,B). Dorsally, both blood and lymphatic vessels were reduced, followed by stasis in vascular density through day 14 (Fig. 4A). Similar findings were observed ventrally (Fig. 4B). No discernible corneal NV was observed, despite the debridement of the entire limbus.

### Deep debridement of dorsal half of the limbal plus whole corneal epithelium (HL + C)

**Figure 5:**
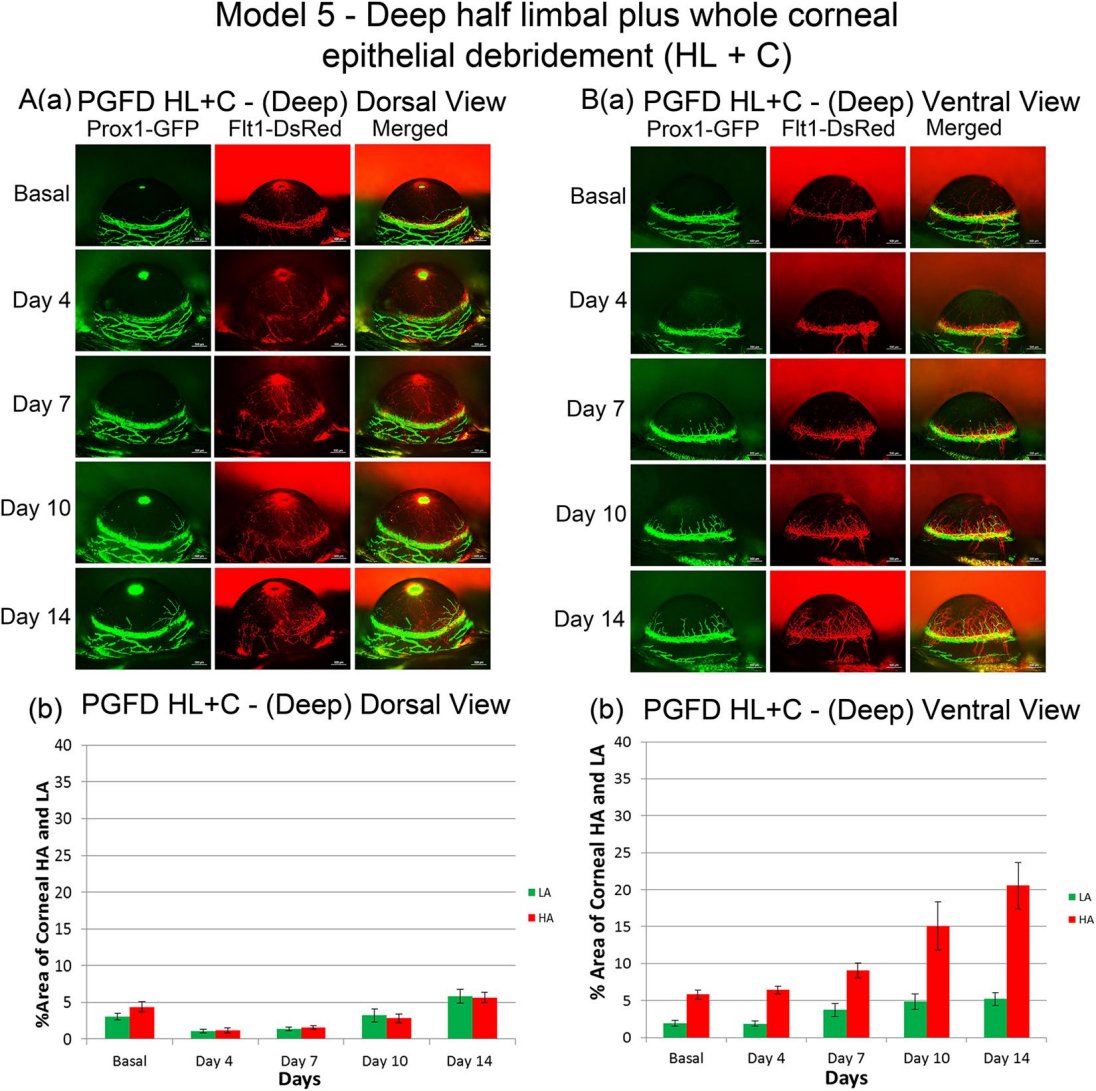
Axio Zoom images of mouse eyes as well as quantitated areas of corneal HA and LA after deep debridement of the dorsal half of the limbal plus whole corneal epithelium. From the dorsal view, both blood and lymphatic vessels regrew toward but not beyond the baseline. The ventral view exhibited growth of both blood and lymphatic vessels surpassing the baseline from day 7 and onward. ([**A**] comparison of dorsal HA on day 0 and day 14 (p = 0.6765), comparison of dorsal LA on day 0 and day 14 (p = 0.1824); [**B**] comparison of ventral HA on day 0 and day 14 (p = 0.0054), comparison of ventral LA on day 0 and day 14 (p = 0.4274). *n* = 5).

In Model 5, deep debridement of dorsal half of the limbal epithelium and the whole corneal epithelium was applied (Fig. 5A,B). Dorsally, both blood and lymphatic vessels were reduced on day 4 and regrowth toward the baseline level was observed by day 14. Ventrally, increases in corneal blood and lymphatic vessels were observed on day 7 and thereafter. Deep debridement of the dorsal half of the limbus along with the whole cornea led to limited corneal NV on the dorsal side compared to the ventral side.

### Comparison of blood and lymphatic vessel growth among the five injury models

**Figure 6:**
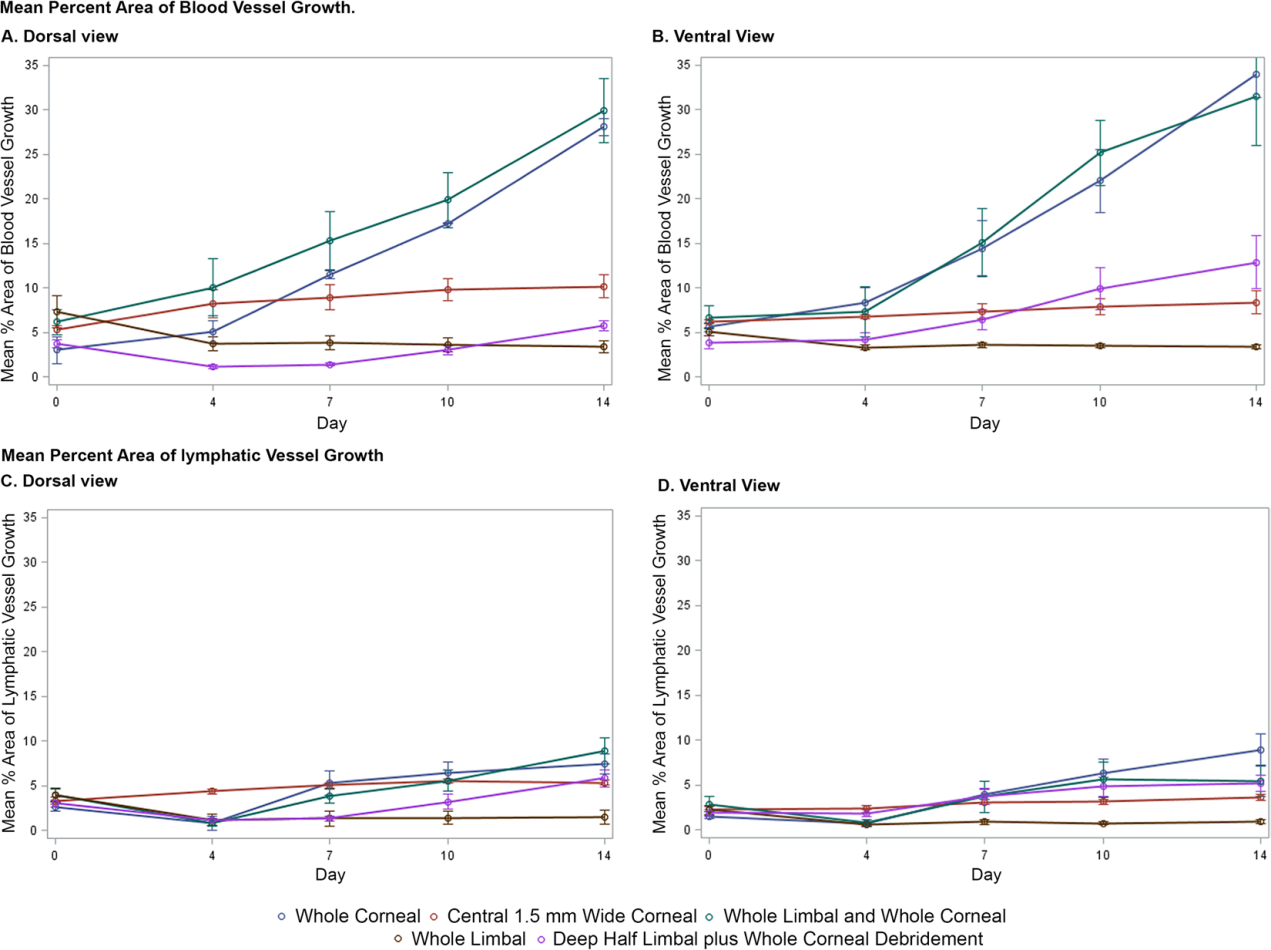
Mean percent area of blood and lymphatic vessels in five debridement models over a 14-day period.

The trends of blood and lymphatic vessel growth can be observed based on the changes in the percent area of vessels from the five injury models (Fig. 6). From both the dorsal and ventral views, relative to the whole corneal epithelial model, the extent of blood and lymphatic vessel growth in the whole limbal, central 1.5 mm wide corneal, and deep half limbal plus whole corneal epithelial debridement models is reduced significantly.

#### Mean percent area of blood vessel growth

Dorsally, the percent area of angiogenic growth in the whole corneal debridement model was 8.60% greater than that in the whole limbal debridement model (P = 0.0012) and 4.52% greater than that in the central 1.5-mm wide corneal debridement model (P = 0.043) (Fig. 6A,B). Additionally, the percent area dorsally was almost 10% greater in the whole cornea debridement model than in the deep hemi-limbal plus whole corneal debridement model (P < 0.001). Ventrally, the percent area angiogenic growth in the whole corneal debridement model was 13.12% greater than that in the whole limbal debridement model (P = 0.0001) and 9.56% greater than that in the central 1.5-mm wide corneal debridement model (P = 0.0014). The percent area ventrally was almost 9.44% greater in the whole cornea debridement model than in the deep hemi-limbal plus whole corneal debridement model (P = 0.0002).

#### Mean percent area of lymphatic vessel growth

Although the percentage area difference in lymphatic growth between the debridement models was statistically significant, the extent was much less profound than that observed for angiogenesis (Fig. 6C,D). Dorsally, the percent area of lymphangiogenic growth in the whole corneal debridement model was 2.67% greater than that in the whole limbal debridement model (P = 0.0023). The percent area dorsally was only 1.65% greater in the whole cornea debridement model than in the deep hemi-limbal plus whole corneal debridement model (P = 0.03). Ventrally, lymphatic growth was 3.20% greater in the whole cornea debridement model than in the whole limbal debridement model (P = 0.0009).

### Debridement of the whole corneal epithelium in *CDh5-CreERT2VEGFR2lox/PGFD* mouse

**Figure 7:**
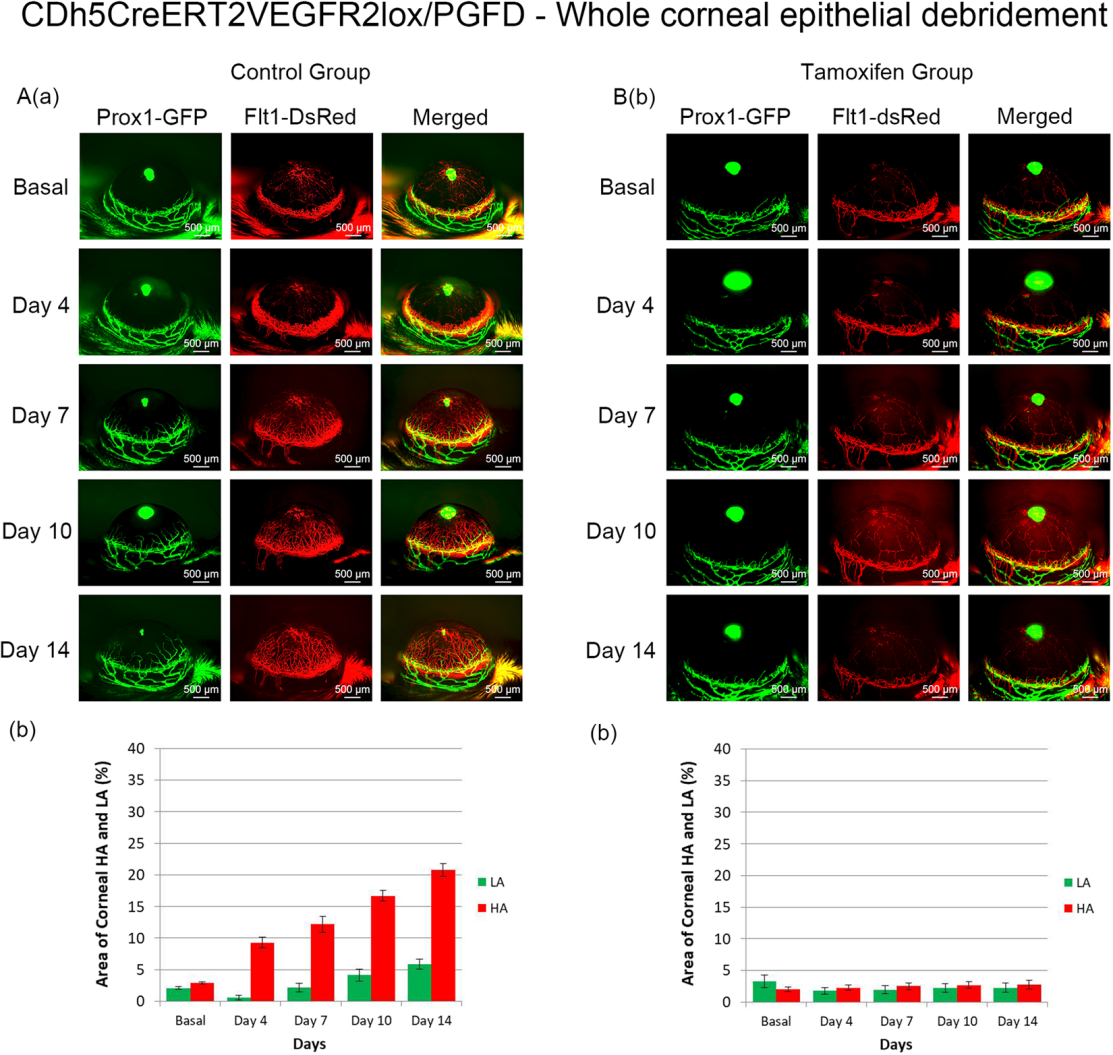
Axio Zoom images of mouse eyes as well as quantitated areas of corneal HA and LA after whole corneal debridement in CDh5-CreERT2VEGFR2lox/PGFD mice. Significant HA and LA were observed in the control group from Day 4 and Day 10, respectively ([**A**] dorsal view, *n* = 4). Meanwhile, corneal HA and LA in response to whole corneal epithelium debridement were abolished on days 4, 7, 10, and 14 in tamoxifen-treated CDh5-CreERT2 VEGFR2lox/PGFD mice ([**B**] dorsal view, *n* = 3).

Model 2 (whole corneal scraping) was also applied in a VEGFR2-knockout line of PGFD mice (Fig. 7) with and without tamoxifen treatment to conditionally delete VEGFR2. The control group exhibited injury-induced corneal HA and LA. Neither injury-induced corneal HA nor LA was observed after conditional deletion of VEGFR2.

## Discussion

### The corneal and limbal environment

The human cornea is composed of five layers: (1) epithelium, a multi-layered milieu consisting of 5–6 stratified nonkeratinized epithelium of 40–50 µm; (2) Bowman’s membrane, composed of fine randomly arranged collagen fibers, and absent in mice; (3) stroma, a precisely organized arrangement of dense collagen fibrils that consists of the greatest bulk of the corneal thickness; (4) Descemet’s membrane, a thick acellular basement of collagen IV that is absent in mice; and (5) endothelium^21^. The corneal epithelium is home to a vast array of molecules that maintain a delicate balance between a pro-(lymph)angiogenic and anti-(lymph)angiogenic state, favoring the avascular state in the healthy cornea. The basement membrane of the corneal epithelium also plays a major role in the production of antiangiogenic factors. For instance, endostatin, which imposes its antiangiogenic effects in the VEGF pathway and thereby halts endothelial cell proliferation, is produced from collagen type XVIII found in the extracellular matrix of the basement membrane^22^. Other antiangiogenic factors, like thrombospondin (TSP)-1 and -2, are also found in the basement membrane and have been shown to regulate corneal avascularity^23^. There is a wealth of studies that describe corneal epithelial factors, as summarized in detail by our group previously^1^. The tear film overlying the corneal epithelium has also been shown to harbor growth factors, like epidermal growth factor, which not only promotes epithelial regeneration, but may be important for angiogenesis^24^. To assess the association between debridement of the cornea and vascular infiltration, we used a corneal rust ring remover to debride the corneal epithelium. Specifically, the AlgerBrush II, marketed by Latham & Phillips Ophthalmic, was used for debridement, as it has a pressure-sensitive clutch that helps prevent penetration into the corneal stroma. During corneal and limbal debridement, significant care was practiced not to confound results by damaging the stroma, which contains widely distributed keratocytes, a major contributor to wound healing and angiogenesis suppression^25^.

The limbus, the second region of interest in this study, is the transition zone between the avascular cornea and the conjunctiva. Stem cells of the corneal epithelium are currently thought to reside in the limbal basal epithelium, specifically in the Palisades of Vogt, which are infoldings in the limbus that greatly increase the surface area of this putative stem cell niche^26^. These stem cells divide and differentiate into transient amplifying cells, which migrate centrally and replenish naturally sloughed corneal cells or corneal defects. While the involvement of anti-/pro-angiogenic factors in the limbus is unclear, study of the limbal niche is vital to determining the full picture of (lymph)angiogenic privilege, as there is a widely documented association of neovascularization in clinical limbal stem cell deficiency (LSCD). For example, chemical or thermal burns of the limbus, contact lens-related hypoxia, Steven-Johnson syndrome, and congenital aniridia are diseases that cause LSCD and often present with severe neovascularization of the cornea. Furthermore, early studies of limbal stem cells showed that a total limbal deficiency model in rabbits exhibited delayed wound healing accompanied by increased corneal vascularization and conjunctivalization. While the mechanism is not fully known, the limbus is suspected to act as a physiologic or physical barrier against invading vessels. As the home of stem cells that replenish corneal epithelial cells, the limbus may act as a barrier to neovascularization by preventing conjunctival overgrowth into the cornea. Our present study used debridement models, three of which specifically removed the limbus, to observe the progression of (lymph)-angiogenesis over a 2-week period. Importantly, deep debridement of the limbus in Model 5 was performed until limbal blood vessels were no longer seen via fluorescence microscopy, allowed by the use of transgenic mice that express intrinsically fluorescent vessels. An understanding of the limbal anatomy, in which the limbal vasculature resides in the stroma underlying the stem cell-containing basal epithelium, provides a certain level of confidence that limbal stem cells were debrided. Our preliminary confocal microscopic data from ABCB5 antibody staining in PGFD mouse eyes demonstrated the complete debridement of limbal blood vessels and limbal stem cells according to the different injury models. However, further studies with immunostaining for reliable limbal stem cell markers–including ABCB5^27^, P63a^28^, ABCG2^29^, cytokeratin15^30^, cytokeratin14^31^, cytokeratin7^32^, and frizzled7^33^–are warranted to accurately assess the extent of injury.

Another consideration beyond the scope of this study is the heterogeneity of the basement membrane of the cornea, which again plays a significant role in (lymph)angiogenic privilege. The composition of the membrane changes drastically during postnatal development and may differ significantly in the central region of the cornea compared to regions closer to the limbus^34^. Therefore, there may be differences beyond our current understanding between various injury models, in which different areas of the cornea were debrided.

### Corneal and limbal debridement in fluorescent *Prox1-GFP/Flt1-DsRed* mice

In the last few decades, many pro- and anti-(lymph)angiogenic factors have been identified and extensively studied in the context of important pathologies, including tumor metastasis. Many of these molecules have also been clinically investigated as potential targets or agents for chemotherapy. Amidst this excitement, the cornea has been a crucial tissue model for vascular studies, largely due to its baseline avascular state and experimental accessibility for vascular growth induction.

Compared to the visible blood vessels, lymph vessels have posed a greater challenge for investigations of these pro- and anti-(lymph)angiogenic factors, as they require separate imaging techniques due to their transparency. Lymphangiography, lymphoscintigraphy, magnetic resonance lymphangiography (MRL), computed tomography (CT), fluorescence microlymphangiography (FML) and near-infrared fluorescence (NIRF), also termed indocyanin green (ICG) lymphography, are among the *in-vivo* methods used to aid the visualization of lymph vessels^35,36^. Other imaging techniques that have emerged recently include optical coherence tomography (OCT) and optical frequency domain imaging (OFDI), which are non-invasive and do not require exogenous contrast agents^37^. However, not all of these modalities are readily available or commonly used for murine model investigations because of their limitations and difficulties in implementation. Intrastromal injection of fluorescein to detect corneal lymphatic vessels is one technique that has been proposed by Le *et al*. for implementation in murine models^38^. Furthermore, recent advances in the use of fluorescent protein gene expression in transgenic animals have aided studies of these vessels^6,35^. However, despite a growing wealth of knowledge and understanding of (lymph)angiogenesis, the molecular underpinnings of the unique, avascular phenotype of the cornea, as well as its drastic changes in the diseased state, have not been completely explained^5,23,39,40^. The PGFD transgenic mouse used in the present study permits the direct, temporal observation of blood and lymphatic vessels in the eyes, even with subject manipulation. By observing blood and lymphatic vessels simultaneously, we can better understand the processes of angiogenesis and lymphangiogenesis, as well as how they may interact or develop in tandem^41,42^.

Previously, studies demonstrated that neither debridement of the central cornea nor bulbar conjunctiva results in significant vessel growth in the mouse cornea, but that of the total cornea, as well as the bulbar conjunctiva plus the total cornea, does^42^. Substantial corneal debridement is required to induce NV in the mouse cornea, and the corneal epithelium may, therefore, be largely responsible for maintaining corneal avascularity. It has been a long-standing idea that the avascularity of the cornea is also attributable to the limbus^3,43–45^. The present *in vivo* study analyzed the response to debridement of both the limbus and cornea in a step-wise fashion to assess the relative contributions of each epithelia to corneal avascularity. Notably, most models showed fluorescence above the pupil caused by lens epithelial cells that also intensely express Prox1-GFP^41^. In Models 1, 2, 4, and 5, the persistent fluorescein-labeled areas above the pupil presumably result from incomplete pupillary constriction, and thus excitation of the lens.

Angiogenic and lymphangiogenic privilege can largely be interrupted by debridement of the whole cornea alone (Fig. 2A,B). On the other hand, debridement of the limbus did not yield any neovascularization within the 14-day experimental time course. In fact, debridement of the whole limbus did not induce NV beyond a 5% corneal area (Fig. 4A,B). The percent-area difference in angiogenesis in the whole cornea vs whole limbus debridement was 8.61% dorsally (P = 0.0012) and 13.12% ventrally (P = 0.0001). The percent-area difference in lymphangiogenesis in the whole cornea vs whole limbus debridement was 2.67% dorsally (P = 0.0023) and 3.20% ventrally (P = 0.0009). The progression of the (lymph)angiogenic process is summarized in Fig. 6, which graphically represents the changes in vessel growth among the debridement models. These comparisons indicate a far greater role of the corneal epithelium, compared to the limbal epithelium, in the vascular profile of the cornea.

Model 5 involves debridement of the epithelium with deep limbal blood vessels, and thus likely the limbal stem cells, of only the dorsal aspect. The ventral aspect of the limbus was spared from debridement. Earlier studies comparing partial and full-thickness limbal epithelial removal showed that the extent of neovascularization correlates with the extent of limbal damage^43^. However, the results from this model showed that within a 14-day span, no neovascularization or conjunctivalization occurred from the dorsal aspect. Meanwhile, neovascularization was observed on the non-injured ventral aspect, albeit to a significantly reduced extent compared to that after cornea-only debridement. This demonstrated that the corneal epithelial debridement may have disrupted (lymph)angiogenic privilege throughout the cornea, resulting in ventral neovascularization despite an intact ventral limbus. It is conceivable that no neovascularization was observed on the dorsal aspect due to the removal of existing vasculature during the debridement process, which may have delayed (lymph)angiogenesis. This may mark a differentiating point from Chen and Tseng’s full-thickness limbal debridement model, which suggested that stem cell loss is a precondition for abnormal healing and neovascularization^43^. Further research is required to characterize the molecular profile of the corneal and limbal epithelium after these insults.

A comparison of the results of partial corneal versus whole corneal debridement indicates that there may be a positive correlation between the area of corneal injury and NV. Debridement of the whole cornea (model 2) yielded a 4.52% greater area of angiogenesis dorsally and 9.56% ventrally than partial debridement (model 1). It is conceivable, therefore, that an increased area of corneal debridement may yield a greater extent of vascular invasion due to a larger disturbance of homeostatic factors that suppress vascular growth. In fact, Cursiefen *et al*. demonstrated that a 2-mm-diameter area of debridement of the corneal epithelium does not result in neovascularization^46^. The diameter of the corneal epithelium removed in Model 2 of this study was 3.10 +/− 0.3 mm, suggesting a possible critical point at which neovascularization begins to occur. However, this suggestion warrants additional experiments for confirmation.

In Models 2, 3, and 5, we observed progressive angiogenesis through day 14. In experiments that involved alkali corneal burns in Prox1-GFP/Flk1::myr-mCherry mice, robust (lymph)angiogenesis was seen from days 3 to 7 with very little growth and branching occurring beyond this period^41^. This was consistent with the current understanding that epithelial wound closures typically occur by day 5, as demonstrated previously by our group through monitoring of corneal re-epithelialization rates in mice after laser keratectomy^47^. The discrepancy in angiogenesis progression between these experiments may be a result of an intrinsic difference between the way chemical and mechanical debridement impact the angiogenic/anti-angiogenic balance in the epithelium. Alkali wounds typically induce a rapid and intense immune response and spare the basal cells in the basement membrane^48^. Meanwhile, mechanical debridement does not guarantee protection of basement membrane integrity. Thus, the extent to which the pro-/anti-(lymph)angiogenic balance is disturbed may differ considerably between the two methods, as well as other procedural protocols. Several other studies demonstrated earlier completion of corneal re-epithelization after injury, which suggests that wound extension and different debridement methods may yield different healing durations^49–51^. Our observation of ongoing neovascularization for up to 14 days, significantly beyond the time span of corneal re-epithelization, suggests that the balance of pro-/anti-(lymph)angiogenic factors continues to be perturbed even in a seemingly healthy state of the cornea. In order to rule out additional keratitis as a cause of neovascularization, we also monitored epithelial recovery via fluorescein studies. Our preliminary data demonstrated that the corneal epithelium is healed by 2–7 days, depending on the debridement model, suggesting that neovascularization is occurring in the absence of ongoing keratitis or gross epithelial disruption.

Importantly, not all corneal wounds clinically result in neovascularization. In fact, most wounds, such as that seen after excimer laser refractive surgery, occur without it, as the corneal epithelium and stroma undergo wound healing while suppressing angiogenesis to preserve corneal transparency. Some studies have also shown murine wound models that do not yield neovascularization^46^. For example, Cursiefen *et al*. showed that cauterizing the corneal bed does not result in significant vascular development, nor does a 2-mm-diameter de-epithelialization of the central cornea. However, cautery after de-epithelialization led to cautery-induced neovascularization, demonstrating the importance of an intact epithelium in maintaining avascularity. In the avascular wound healing of the cornea, there is increased expression of other anti-angiogenic factors as well, including sflt-1, sVEGFR-2, angiostatin, endostatin, and pigment epithelium-derived factor, while angiogenic factor expression is suppressed^52^. Among these factors, matrix metalloproteinases (MMPs) and tissue-inhibitors of matrix metalloproteinases (TIMPs) have been implicated as counteracting anti-angiogenic and pro-angiogenic regulators, becoming pivotal factors in the delicate balance of vascularization and avascularity. The delicate balance of these factors plays an essential role in inhibiting neovascularization in the context of epithelial injury. However, several inflammatory, infectious, degenerative, and traumatic corneal disorders have been shown to cause (lymph)angiogenesis during wound healing, including the aforementioned causes of LSCD. It is currently thought that the corneal and limbal milieu, responsible for maintaining a molecular profile that favors angiogenic suppression, is disrupted in these corneal or limbal injuries. The exact reasoning behind disparities in various injury models remains a great challenge due to the cellular heterogeneity of the cornea. We have also yet to elucidate the expression gradient of angiogenic and anti-angiogenic factors throughout the cornea, which creates an obstacle in corneal wound model comparisons. However, individual wound models provide an important piece to a larger, complex picture involving corneal angiogenic privilege and wound healing. In the current study, we provided five models of debridement, each of them interrupting the corneal and limbal environment in a step-wise fashion to demonstrate the gross contributions of the limbus and cornea to (lymph)angiogenic privilege using a transgenic mice model.

This temporo-spatial observation of corneal neovascularization was enabled by a dual fluorescent reporter transgenic reporter line. PGFD mice circumvent the need for tissue preparation and thus the need to sacrifice mice between each observation point, allowing for the use of fewer mice and elimination of individual sample variations. This study demonstrates another utility of these mice—cross-breeding with a conditional VEGFR2 knockout model (CDh5-CreERT2VEGFR2lox/PGFD) to observe the importance of a well-studied receptor in vascular growth. We were able control the conditional deletion of VEGFR2 with tamoxifen injections, and with the loss of VEGFR2 in vascular endothelial cells NV was no longer observed following corneal debridement (Fig. 7A,B). This experiment suggests an important role of VEGFR2 in injury-induced neovascularizarion, and identifies a potential opportunity to study VEGFR2 in a therapeutic context. In theory, PGFD mice can be bred with other knockout mice to enable a variety of experimental models for the study of hemangiogenic and lymphangiogenic processes in the cornea and other organ systems.

## Conclusion

Via simultaneous *in vivo* imaging of (lymph)angiogenesis in the cornea of live PGFD mice, the present study characterized the patterns of neovascularization induced by various epithelial debridement models involving the limbus and cornea. The patterns of vessel growth in these injury models suggest a major role of corneal epithelial factors in maintaining angiogenic and lymphangiogenic privilege, as well as an inadequacy in hypotheses suggesting that the limbus acts as a barrier to invasion by peri-corneal vessels. Overall, these findings will aid future research to identify key players in corneal neovascularization and eventually to develop therapies targeting pathologies including limbal stem cell deficiency and graft rejections that are associated with this vascular phenomenon.

## Supplementary information

Supplementary Dataset 1

Author statement
