# Supplementary material for: Application of corneal injury models in dual fluorescent reporter transgenic mice to understand the roles of the cornea and limbus in angiogenic and lymphangiogenic privilege
Source: Sci Rep. 2019 Aug 23;9:12331. doi: 10.1038/s41598-019-48811-z (PMC6707148; doi:10.1038/s41598-019-48811-z)
Supplement: Supplementary file 1 — Supplementary Dataset 1 [file 41598_2019_48811_MOESM1_ESM.pdf]

Application of corneal injury models in dual fluorescent reporter transgenic mice to understand the roles of the cornea and limbus in angiogenic and lymphangiogenic privilege

Xinbo Gao, MD, PhD[+], Kai Guo, MD, PhD[+], Samuel M. Santosa, MD, Mario Montana, MD, Michael Yamakawa, BA, Joelle A. Hallak, PhD, Kyu-Yeon Han, PhD, Susan J. Doh, BA, Mark I. Rosenblatt, MD, PhD, MBA, Jin-Hong Chang, PhD* and Dimitri T. Azar, MD, MBA*

Department of Ophthalmology and Visual Sciences, Illinois Eye and Ear Infirmary, College of Medicine, University of Illinois at Chicago, Chicago, Illinois.

[+]equal contribution

*Corresponding authors: Dimitri T. Azar, MD, MBA (dazar@uic.edu); Jin-Hong Chang, PhD (changr@uic.edu)
Phone: (312) 413-5590, Fax: (312) 996-7770

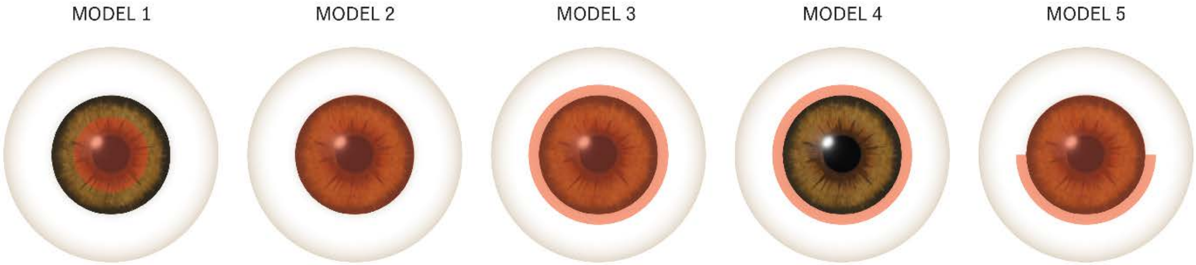

Supplemental Figure 1: Diagrams of the five different models of corneal and/or limbal injury by debridement. Model 1: debridement of a 1.5-mm-diameter circle in the center of the corneal epithelium; Model 2: debridement of the whole corneal epithelium (WC); Model 3: superficial debridement of the whole limbal plus whole corneal epithelium (superficial L+C); Model 4: superficial debridement of the whole limbal epithelium (WL); and Model 5: deep debridement of half the limbal epithelium and the whole corneal epithelium (HL+C).
