# Supplementary material for: Application of corneal injury models in dual fluorescent reporter transgenic mice to understand the roles of the cornea and limbus in angiogenic and lymphangiogenic privilege
Source: Sci Rep. 2019 Aug 23;9:12331. doi: 10.1038/s41598-019-48811-z (PMC6707148; doi:10.1038/s41598-019-48811-z)
Supplement: Supplementary file 2 — Author statement [file 41598_2019_48811_MOESM2_ESM.docx]

**Method statement and guidance:**

All animal experiments were done in accordance with guidelines and approved by IACUC at the University of Illinois at Chicago.

**Acknowledgements**

Publication of this article was supported by National Institutes of Health grants EY10101 (D.T.A.), I01 BX002386, EY01792, and EY027912 (MIR); the Eversight, Midwest Eye Bank Award (J.H.C); and an unrestricted grant from Research to Prevent Blindness, New York, NY.

**Author information**

**Author notes**

Xinbo Gao, MD, PhD

Kai Guo, MD, PhD

These authors contributed equally to this work.

**Affiliations**

Department of Ophthalmology and Visual Sciences, Illinois Eye and Ear Infirmary, College of Medicine, University of Illinois at Chicago, Chicago, IL, USA

**Contributions**

X.G., K.G., M.I.R, J.H.C., and D.T.A. designed the experiments. X.G., K.G., and J.H.C carried out all the experimental work. M.Y., J.A.H., D.T.A., M.I.R., J.H.C., S.M.S., M.M., and K.Y.H. analyzed the data and wrote and reviewed the manuscript.

**Competing interests**

The authors declare no competing financial interests.

**Corresponding authors**

Correspondence to Dimitri T. Azar and Jin-Hong Chang
